# Factors Associated with Complete Home Smoking Ban among Chinese Parents of Young Children

**DOI:** 10.3390/ijerph13020161

**Published:** 2016-01-26

**Authors:** Kaiyong Huang, Hailian Chen, Jing Liao, Guangmin Nong, Li Yang, Jonathan P. Winickoff, Zhiyong Zhang, Abu S. Abdullah

**Affiliations:** 1 School of Public Health, Guangxi Medical University, Nanning 530021, Guangxi, China; huangka0319@sina.com (K.H.); yangli8290@hotmail.com (L.Y.); rpazz@163.com (Z.Z.); 2 School of Information and Management, Guangxi Medical University, Nanning 530021, Guangxi, China; chlq869007621@163.com; 3 Department of Pediatrics, The First Affiliated Hospital of Guangxi Medical University, Nanning 530021, Guangxi, China; gxlmd@126.com (J.L.); ngm8525@hotmail.com (G.N.); 4 MGH Center for Child and Adolescent Health Research and Policy, Harvard Medical School, Boston, MA 02115, USA; JWINICKOFF@PARTNERS.ORG; 5 Global Health Program, Duke Kunshan University, Kunshan 215347, Jiangsu Province, China; 6 Duke Global Health Institute, Duke University, Durham, NC 27710, USA; 7 School of Medicine, Boston Medical Center, Boston University, Boston, MA 02118, USA

**Keywords:** environmental tobacco smoke, home smoking ban, children, China

## Abstract

(1) *Background*: The home environment is a major source of Environmental Tobacco Smoke (ETS) exposure among children especially in early childhood. ETS exposure is an important health risk among children and can cause severe and chronic diseases, such as asthma, bronchitis, and premature death. However, ETS exposure at home has often been neglected in the Chinese families. Identification of factors that facilitate or otherwise hamper the adoption of home smoking ban will help in the design and implementation of evidence-based intervention programs. This study identifies factors correlated with home smoking bans in Chinese families with children. (2) *Methods*: A cross-sectional survey of parents living in Nanning city, Guangxi Province, China with at least one smoker and a child in the household was conducted between September, 2013 and January, 2014. A Chi-square test was used to compare categorical variables differences between the parents who had home smoking bans and those with no home smoking ban. Multiple logistic regression analyses were used to identify factors correlated with home smoking bans. (3) *Results*: 969 completed questionnaires were collected with a response rate of 92.29% (969/1050). Of the respondents (*n* = 969), 14.34% had complete home smoking bans. Factors that were associated with home smoking bans were: having no other smokers in the family (*OR* = 2.173), attaining education up to high school (*OR* = 2.471), believing that paternal smoking would increase the risk of lower respiratory tract illnesses (*OR* = 2.755), perceiving the fact that smoking cigarettes in the presence of the child will hurt the child’s health (*OR* = 1.547), believing that adopting a no smoking policy at home is very important (*OR* = 2.816), and being confident to prevent others to smoke at home (*OR* = 1.950). Additionally, parents who perceived difficulty in adopting a no smoking policy at home would not have a home smoking ban (*OR* = 0.523). (4) *Conclusions*: A home smoking ban is not widely adopted by families of hospitalized children in Guangxi Province, China. To protect the health of children, there is a need to develop and test interventions to promote home smoking bans. Factors identified as predictors of home smoking ban should be considered in the design of interventions.

## 1. Introduction

Secondhand Smoke (SHS) is defined as “the combination of smoke emitted from the burning end of a cigarette or other tobacco products and smoke exhaled by the smoker” [1]. Thirdhand smoke (THS) is a complex phenomenon resulting from residual tobacco smoke pollutants that adhere to the clothing and hair of smokers and to surfaces, furnishings, and dust in indoor environments [2]. Protano and Vitali proposed that “environmental tobacco smoke (ETS)” be used as a more inclusive term to describe any tobacco smoke exposure outside of active smoking [2], to describe SHS and THS. ETS is one of the most common and important sources of indoor air pollution in the home environment. An estimated 600,000 deaths each year worldwide are caused by ETS exposure, accounting to more than 1% of all deaths [3]. ETS exposure increases childrens’ risk of having a carotid atherosclerotic plaque [4], respiratory tract infections, otitis media, asthma, and sudden infant death syndrome [5]. Eliminating indoor smoking practices in households can greatly protect children from the health effects of ETS exposure [6]. Study showed that the impact of at-home-smoking practices on children’s ETS exposure is highlighted by the significant and progressive increases in urinary cotinine levels from children not living with smoker(s) to children living with smoker(s) who do not smoke at home to children living with smoker(s) who only smoke at home when the child is not there, and finally to children living with smoker(s) who smoke at home even if the child is in [7]. Implementing smoke-free policies in work places and public places have been proven to contribute to improving the public’s knowledge of the harm of SHS exposure and changes in their smoking behavior, which could lead to increased adoption of voluntary home smoking bans [6,8].

China is the largest producer and consumer of tobacco globally, with more than 350 million smokers [9] and 740 million non-smokers passively exposed to ETS exposure, including 180 million children under 15 years old [10,11]. ETS exposure is associated with an estimated 100,000 deaths annually in China [10]. The 2010 China Global Adult Tobacco Survey (GATS) reported that 67% of non-smokers are exposed to ETS in their homes, 73% in public places, 63% in work places, 58% in government buildings, and 37% in schools [12]. Household ETS exposure rates in China were higher than in the United States [13], Korea [14], and Mexico [15]. Results from China National Behavior Monitoring, indicated the prevalence of ETS exposure in China persisted without any decline from 2002 to 2010 [8,16]. Since 2010, smoking bans policies have been gradually implemented in public areas including government buildings, public transportation, healthcare facilities, schools, workplaces, bars, and restaurants in China [17]. However, ETS exposure at home has often been neglected, despite being a major source of exposure to tobacco smoke among children, especially preschool children, who spent much of their time at home [18]. The low rates of home smoking ban in China, ranging between 6.3%–26% [19,20], indicate the fact that a large proportion of Chinese children are exposed to ETS at home, underscoring the need to identify factors that are associated with adopting home smoking bans. A home smoking ban is an effective, feasible, and relatively new measure to prevent ETS exposure among children. Studies have shown that implementation of a home smoking ban in families with a smoker will greatly reduce ETS (or SHS) exposure among children [21,22]. It is important to identify factors that facilitate or hamper the adoption of home smoking bans in order to develop intervention strategies to reduce ETS exposure for children in the home environment. Nevertheless, there are few studies that reported factors influencing the implementation of complete home smoking ban in China. 

This study estimated the prevalence of home smoking bans in Guangxi Province, China, through a quantitative study of parents whose children were hospitalized in two major hospitals. The study identified factors correlated with hospitalized children’s home smoking bans, and provided recommendations for implementing measures that promote home smoking bans. The home smoking ban considered in this study applies not only to parental smoking, but also to smoking by other cohabitants and visiting guests. 

## 2. Methods

### 2.1. Design and Sample

A cross-sectional study was conducted from September 2013 to January 2014 among children’s parents attending in the pediatric in-patient departments of two grade-three hospitals in Nanning, Guangxi province: First Affiliated Hospital of Guangxi Medical University and the Nanning Maternal and Child Health Hospital. Grade 3 hospitals are general or comprehensive hospitals at the national, provincial, or city level (>500 beds) and has most of the medical and surgical specialties. Parents of hospitalized children in these two hospitals were approached for a face-to-face interview. A standardized Chinese language questionnaire was used to collect data. The questionnaire was developed with reference to the questionnaires previously used and validated by the investigato’s team in China [23]. The modified questionnaire was pre-tested with 10 parents at Guangxi Medical University hospital for clarity and cultural acceptability. This required minor modification before finalization of the questionnaire. Trained research assistants conducted face to face interviews, using a structured questionnaire, to collect data from parents. Checks were carried out to ensure all the questions had been answered and incase of any discrepancies (unclear answers, unfinished questions, and/or logistic errors), the investigators contacted the individual by telephone or requested a re-interview when necessary. As a token of appreciation, each participant received a small gift. The study protocol was approved by the Ethical Committee of Guangxi Medical University (Number: 2013/ethics/SPH/03; dated, 15 March 2013) and informed consent was received from all individuals who agreed to participate in the study. 

### 2.2. Measures

Data collection included demographic information (gender, age, ethnicity, education, income, and children’s age) and smoking behavior (smoker, non-smoker). Questions asked to measure attitudes and practices about smoking policy and home smoking included: “How important is it for you to adopt a no smoking policy at home?” with response categories of “Very important, Important, Unsure, A little important, Not at all important”; “How much difficulty do you think you’ll have adopting a no smoking policy at your home?” with response categories of “Very difficult, Difficult, Unsure, A little difficult, Not at all difficult”; “How confident are you that you would not allow others to smoke at your home?” with response categories of “Very confident, Confident, Unsure, A little confident, Not at all confident”; “How concerned are you that smoking cigarettes in the presence of your child will hurt your child’s health?” with response categories of “Very concerned, Concerned, Unsure, A little concerned, Not at all concerned”; “Have you ever heard of third hand smoke” with response categories of “Yes or No”, and other questions on “My being a smoker gets in the way of my being a parent”, “Smoking in enclosed public places should be prohibited”, “Breathing air in a room today where people smoked yesterday can harm the health of infants and children”, and “Paternal smoking increases the risk of lower respiratory tract illnesses such as pneumonia in exposed children”, with response categories of “Strongly agree, Agree, Disagree and Strongly disagree”. 

During analysis, responses were condensed into two categories each of “Very important/Important” and “Unsure/A little important/Not at all important”, “Very difficult/Difficult” and “Unsure/A little difficult/Not at all difficult”, “Very confident/Confident” and “Unsure/A little confident/Not at all confident”, “Very concerned/Concerned” and “Unsure/A little concerned/Not at all concerned”, “Strongly agree/Agree” and “Disagree/Strongly disagree” [24]. We defined “smoker” as those who smoked at the time of the survey, and defined “non-smoker” as those who never smoked or had quit smoking at the time of survey [25].

### 2.3. Analyses

Two members of the research team coded each questionnaire and entered all data in Epidata 3.1, and carried out data consistency checks. To examine the differences between the parents who had a home smoking ban and those without home smoking ban, a Chi-square test was performed. The factors with significant difference in chi-square test were included in multiple logistic regression analyses. The multiple logistic regression model was adjusted for age and sex. A *p*-value of <0.05 (two-tailed) was considered statistically significant. SPSS (version 13.0, IBM, New York, NY, USA) was used to conduct all statistical analyses.

## 3. Results

### 3.1. Demographic and Other Characteristics Information

A total of 969 participating parents successfully completed the questionnaires representing a response rate of 92.29% (969/1050). Of the respondents (*n* = 969), 68.83% were female, the majority (89.88%) were under 45 years old, and 53.04% were ethnic Han. Only 18.99% of the respondents attained a college or higher level of education and 56.86% only received the nine-year compulsory education. The unemployment rate among the respondents was 42.72%. Almost half (49.74%) of the respondents had an annual household income below 3000CNY (about $490) per month. 74.30% children were under five years old. Overall, 16.82% of the respondents were smokers, and 56.76% had one or more smokers living in the same household. (Table 1).

**Table 1:** Characteristics of participating parents (*N* = 969).

Variables	*N* (%)
**Gender**	
Male	302 (31.17%)
Female	667 (68.83%)
**Age**	
18–30	406 (41.89%)
31–44	465 (47.99%)
Above 45	98 (10.12%)
**Ethnicity**	
Han	514 (53.04%)
Other ethnicities	455 (46.96%)
**Education**	
Below high school	551 (56.86%)
High school graduate	234 (24.15%)
College or above	184 (18.99%)
**Income per month**	
Below 3000 CNY	482 (49.74%)
3000–6000 CNY	331 (34.17%)
Above 6000 CNY	156 (16.09%)
**Children’s age**	
Under 5 years	720 (74.30%)
5 years or above	249 (25.70%)
**Other smokers in home**	
None	419 (43.24%)
One or more	550 (56.76%)
**Smoking behavior**	
Smoker	163 (16.82%)
Non-smoker	806 (83.18%)
**Having complete home smoking ban**	
Yes	139 (14.34%)
No	830 (85.66%)

### 3.2. Factors Associated with Complete Home Smoking Ban

14.34% (139/969) of the respondents reported that they had complete home smoking bans. Variables that showed significant bivariate associations with complete home smoking bans included: participating parents’ gender (*χ*^2^ = 6.947, *p* = 0.008), education (*χ*^2^ = 26.118, *p* < 0.001), income (*χ*^2^ = 7.209, *p* = 0.027), whether have other smokers at home or not (*χ*^2^ = 37.032, *p* < 0.001), and is the participant a smoker? (*χ*^2^ = 7.776, *p* = 0.006). Compared with participating parents without a complete home smoking ban, participating parents’ attitudes and practices about home smoking ban yielded the following results: “Paternal smoking increases the risk of lower respiratory tract illnesses such as pneumonia in exposed children” (*χ*^2^ = 4.748, *p* = 0.029), “How concerned are you that smoking cigarettes in the presence of your child will hurt your child’s health?” (*χ*^2^ = 75.688, *p* < 0.001), “How important is it for you to adopt a no smoking policy at home” (*χ*^2^ = 10.396, *p* = 0.001), “How much difficulty do you think you’ll have adopting a no smoking policy at your home” (*χ*^2^ = 34.559, *p* < 0.001), and “How confident are you that you would not allow others to smoke at your home” (*χ*^2^ = 37.291, *p* < 0.001) showed significant differences. (Table 2).

**Table 2:** Complete home smoking bans by selected characteristics (*N* = 139).

Variables	Complete Home Smoking Ban *N* (%)	χ^2^	*p*
**Gender**			
Male	30 (3.09%)	6.947	0.008 ^a^
Female	109 (11.25%)		
**Age**			
18–30	51 (5.25%)	1.818	0.403 ^a^
31–44	73 (7.52%)		
Above 45	15 (1.57%)		
**Ethnicity**			
Han	72 (7.43%)	0.101	0.750 ^a^
Other ethnicities	67 (6.91%)		
**Education**			
Under high school	52 (5.37%)	26.118	<0.001 ^a^
High school graduate	45 (4.64%)		
College or above	42 (4.33%)		
**Income**			
Below 3000CNY per month	65 (6.71%)	7.209	0.027 ^a^
3000–6000CNY per month	41 (4.23%)		
Above 6000CNY per month	33 (3.40%)		
**Children’s age**			
Under 5 years	98 (10.11%)	1.227	0.268 ^a^
5 years or above	41 (4.23%)		
**Other smokers at home**			
None	93 (9.59%)	37.032	<0.001 ^a^
More than 1	46 (4.75%)		
**Smoking behavior**			
Smoker	12 (1.24%)	7.776	0.006 ^a^
Non-smoker	127 (13.10%)		
**My being a smoker gets in the way of my being a parent**			
Strongly agree/Agree	130 (13.42%)	0.420	0.517 ^a^
Disagree/Strongly disagree	9 (0.92%)		
**Smoking in enclosed public places should be prohibited**			
Strongly agree/Agree	138 (14.24%)	0.600	0.439 ^a^
Disagree/Strongly disagree	1 (0.10%)		
**Breathing air in a room today where people smoked yesterday can harm the health of infants and children**			
Strongly agree/Agree	127 (13.11%)	1.112	0.292 ^a^
Disagree/Strongly disagree	12 (1.23%)		
**Paternal smoking increases the risk of lower respiratory tract illnesses such as pneumonia in exposed children.**			
Strongly agree/Agree	132 (13.62%)	4.748	0.029 ^a^
Disagree/Strongly disagree	7 (0.72%)		
**How concerned are you that smoking cigarettes in the presence of your child will hurt your child’s health?**			
Very concerned/Concerned	105 (10.83%)	75.668	<0.001 ^a^
Unsure/A little concerned/Not at all concerned	34 (3.51%)		
**How important is it for you to adopt a no smoking policy at home**			
Very important/Important	129 (13.31%)	10.396	0.001 ^a^
Unsure/A little important/Not at all important	10 (1.03%)		
**How much difficulty do you think you’ll have adopting a no smoking policy at your home**			
Very difficult/Difficult	29 (2.99%)	34.559	<0.001 ^a^
Unsure/A little difficult/Not at all difficult	110 (11.35%)		
**How confident are you that you would not allow others to smoke at your home**			
Very confident/Confident	84 (8.67%)	3.049	<0.001 ^a^
Unsure/A little confident/Not at all confident	55 (5.67%)		
**Have you ever heard of third hand smoke**			
Yes	31 (3.19%)	3.788	0.052 ^a^
No	108 (11.15%)		

**^a^** Chi-square test.

### 3.3. Multiple Logistic Regression Analysis to Identify Predictors of Having a Complete Home Smoking Ban

As shown in Table 3, factors that were associated with home smoking bans based on the results of the multiple logistic regression analysis included: having no other smokers in the family (*OR* = 2.173), graduating from high school (*OR* = 2.471), believing that paternal smoking would increase the risk of lower respiratory tract illnesses (*OR* = 2.755), perceiving the fact that smoking cigarettes in the presence of child will hurt child’s health (*OR* = 1.547), believing that adopting a no smoking policy at home is very important (*OR* = 2.816) , and being confident to prevent others to smoke at home (*OR* = 1.950). Additionally, parents who perceived difficulty in adopting a no smoking policy at home would not have a home smoking ban (*OR* = 0.523).

## 4. Discussion

In this study, the prevalence of home smoking bans reported by parents of hospitalized children was 14.34%, which is much lower than the reported prevalence of 38% among Chinese Americans [26] and 26% in Shanghai, China [19]. A possible explanation of low home smoking bans in the current study could be that smoke-exposed children may be more likely to be hospitalized. Guangxi, being an underdeveloped area, most of the people were committed to improving their poverty status and many use tobacco to relieve stress or social interactions, and unlikely to take any self-initiated measure to reduce exposure to ETS [27]. Possible explanations are a lack of parental awareness or concern about the negative effects of ETS exposure on children, less-established social norms for protecting children from ETS exposure, and a traditional Chinese culture that reinforces obedience and prevents children from disagreeing with or complaining to parents about smoking [19]. Therefore, we may predict that the implementation rate of household smoking ban of average family in Guangxi is below than the rate at the national or global level.

Demographic information indicated that income and participating parents’ smoking status were significantly related to having a home ban in bivariate, but not multivariate, analyses. Household smoking restrictions were all associated with gender, education, and the existence of other smokers at home in both bivariate and multivariate analyses. The results showed that women were more likely to live in households with complete bans than men (11.25% *vs*. 3.09%), contradicting the findings of an earlier study among Chinese Americans [25]. However, cultural norms that Chinese women are often treated based on stereotypical roles in the household as mother, daughter in-law, wife, or daughter, rather than on their needs and desires as distinct individuals [23], may dictate that women have less control over setting this type of policy in the home, and female non-smokers are more likely than male non-smokers to live with a smoker. This finding requires further exploration but suggests that ETS exposure reduction interventions need to be developed and tested, putting into consideration Chinese cultural norms [25]. At the same time, it is important to empower women to become advocates for a home environment free of the harmful effects of ETS exposure [28]. 

**Table 3:** Logistic regression model to identify predictors of having a complete home smoking ban.

Variables	Adjusted OR	95% CI
**Income per month**		
Below 3000CNY	1.000	Reference
3000–6000CNY	0.867	0.485, 1.547
Above 6000CNY	1.453	0.816, 2.584
**Education**		
Below high school	1.000	Reference
High school graduate	2.471	1.374, 4.445
College or above	1.125	0.646, 1.960
**Smoking behavior**		
Smoker	0.896	0.391, 2.053
Non-smoker	1.000	Reference
**Other smokers at home**		
None	2.173	1.405, 3.361
More than 1	1.000	Reference
**Paternal smoking increases the risk of lower respiratory tract illnesses such as pneumonia in exposed children**		
Strongly agree/Agree	2.755	1.149, 6.607
Disagree/Strongly disagree	1.000	Reference
**How concerned are you that smoking cigarettes in the presence of your child will hurt your child’s health?**		
Very concerned/Concerned	1.547	1.250, 3.495
Unsure/A little concerned/Not at all concerned	1.000	Reference
**How important is it for you to adopt a no smoking policy at home**		
Very important/Important	2.816	1.316, 6.025
Unsure/A little important/Not at all important	1.000	Reference
**How much difficulty do you think you’ll have adopting a no smoking policy at your home**		
Very difficult/Difficult	0.523	0.316, 0.863
Unsure/A little difficult /Not at all difficult	1.000	Reference
**How confident are you that you would not allow others to smoke at your home**		
Very confident/Confident	1.950	1.260, 3.016
Unsure/A little confident/Not at all confident	1.000	Reference

Some researchers have reported that indoor household smoking bans are less common among low-income households, and attributed this relationship to the fact that those with higher incomes had higher educational attainment and were more aware of the hazards of SHS exposure [28,29]. Our study found that household income level was negatively associated with household smoking ban, after considering all the factors in multivariate analyses. Chinese nine-year compulsory education rarely involves knowledge of the health hazards of tobacco, but previous study [30] found that education regarding the impact of SHS exposure on children and adolescents may impact future family practices around smoking in the home. Thus, we obtained that the higher the education levels, the greater possibility of the family tobacco control measures. Our findings which were consistent with the previous study [31] showed that adults and children of Chinese descent living in households with one or more smokers (including the respondent) were less likely to experience home smoking restriction than their counterparts who did not live with smokers. This indicates the need to educate smoker household members with targeted messages about the importance of and need to adopt home smoking bans.

Similar to previous studies [26,32], this study shows that individual awareness of the harms of ETS exposure plays a protective role regarding implementation of home smoking bans. The largest gains in home smoking bans occurred in families with the youngest children. This suggests that campaigns encouraging parents not to smoke around babies have been very effective. These benefits of home smoking bans include reducing ETS exposure among children and adults [7], promoting anti-smoking attitudes among youth, decreasing smoking and smoking uptake in teenagers and increasing quitting [33,34,35,36].

One limitation of the current study is that we recruited parents of hospitalized children from two grade-three hospitals in Guangxi, which may not represent the characteristics of participating parents attending in grade 1 or 2 hospitals, as well as the non-hospitalized population. The grade-three hospital in China is the best hospital with the highest qualified doctors and nurses, greatest medical service and management, best medical quality and safety, most advanced technical level, and efficiency. The grade-three hospital is better than the grade-two hospital, and the grade-two hospital is better than the grade-one hospital in all quality measures. 

## 5. Conclusions

The findings of this study show that home smoking ban is not widely adopted by families of hospitalized children in Guangxi, China, which underscores the fact that many children are exposed to ETS at home. Several factors including parental misconceptions about the harms of smoking and ETS exposure and lack of awareness about the usefulness of home smoking ban was associated with not adopting smoke-free home policies. Tobacco control programs, primarily designed to minimize spread of, and exposure, to ETS, should be used to promote and strengthen the public awareness of added benefits achievable through home smoking restrictions in addition to dispelling their misconceptions about smoking and ETS. 
